# Multi-omics analysis revealed that Jingfang Mixture alleviates inflammation in an OVA/Al(OH)_3_-induced urticaria-like rat model by regulating the AMPK/p38 MAPK/PI3K-Akt pathway associated with the gut-skin axis

**DOI:** 10.3389/fcimb.2026.1973292

**Published:** 2026-10-29

**Authors:** Xiaowen Song, Xuexin Liu, Le Yang, Guangli Yan, Hui Sun, Ye Sun, Guimin Zhang, Ling Kong, Ying Han, Yongxia Guan, Xijun Wang

**Affiliations:** 1 State Key Laboratory of Integration and Innovation of Classic Formula and Modern Chinese Medicine, National Chinmedomics Research Center, National TCM Key Laboratory of Serum Pharmacochemistry, Metabolomics Laboratory, Department of Pharmaceutical Analysis, Heilongjiang University of Chinese Medicine, Harbin, China; 2 State Key Laboratory of Dampness Syndrome, The Second Affiliated Hospital Guangzhou University of Chinese Medicine, Guangzhou, China; 3 State Key Laboratory of Integration and Innovation of Classic Formula and Modern Chinese Medicine, Lunan Pharmaceutical Group Co. Ltd., Linyi, China

**Keywords:** AMPK/p38 MAPK/PI3K-Akt pathway, gut microbiota, gut-skin axis, Jingfang Mixture, multi-omics, traditional Chinese medicine, urticaria

## Abstract

**Background:**

The systemic and relapsing nature of urticaria cannot be fully explained by cutaneous mast-cell activation alone. Whether the gut contributes, and whether a multi-component formula such as Jingfang Mixture (JF) acts through a gut-skin route, have not been tested together at the level of microbiota, metabolites, and skin signaling together.

**Methods:**

Using an OVA (ovalbumin)/Al(OH)_3_-induced urticaria-like allergic inflammation rat model, we integrated 16S rRNA gene sequencing, fecal untargeted metabolomics, and skin DIA (data-independent acquisition)-based quantitative proteomics to investigate the therapeutic mechanisms of JF.

**Results:**

JF suppressed both Th2 (IgE (immunoglobulin E), IL-4 (interleukin-4)) and Th17/pro-inflammatory (IL-6 (interleukin-6), TNF-α (tumor necrosis factor-α), IL-17 (interleukin-17)) responses, improved intestinal barrier-associated abnormalities, and partially reversed gut dysbiosis by restoring SCFA-producing bacteria (*Bacteroidia, Clostridia, Lachnospirales, Oscillospirales*) while reducing *Proteobacteria* and *Enterobacterales*. JF normalized the fecal metabolome, elevating anti-inflammatory metabolites including butyrate, indole, indole-3-acetic acid, docosahexaenoic acid (DHA) and reducing pro-inflammatory mediators such as arachidonic acid, leukotriene B4, secondary bile acids. At the skin level, JF activated AMPK (AMP-activated protein kinase) signaling and suppressed aberrant p38 MAPK (p38 mitogen-activated protein kinase) and PI3K (phosphoinositide 3-kinase)-Akt (protein kinase B) activation. Spearman correlation analysis further revealed that SCFAs (short-chain fatty acids) and indole derivatives were positively correlated with the abundance of AMPK-pathway-related proteins and negatively with p38 MAPK and PI3K-Akt proteins, whereas pro-inflammatory metabolites exhibited inverse correlation patterns.

**Conclusion:**

Collectively, our findings indicate that JF mitigates urticaria-like inflammation, likely through gut microbiota and modulating SCFA, bile acid, tryptophan, and lipid metabolism. This multi-omics evidence suggests that JF may act through the gut microbiota-metabolite-skin signaling axis to confer anti-urticarial benefits. Nevertheless, further causal validation experiments and clinical investigations are warranted.

## 1 Introduction

Urticaria is an immuno-inflammatory skin disorder clinically characterized by transient wheals, intense pruritus, or angioedema. Its complex pathogenesis involves aberrant mast cell activation, Th1/Th2 immune imbalance, IgE- driven hypersensitivity, and release of multiple pro-inflammatory mediators (Kolkhir et al., 2024; Puxeddu et al., 2024). Accumulating evidence suggests that urticaria is not merely a local cutaneous allergic disorder but a systemic disease featured by pervasive immune dysregulation and chronic low-grade inflammation. The Global Burden of Disease Study 2021 reported that the global disease burden of urticaria has continuously increased over the past three decades and is projected to keep rising in the future (Cao et al., 2025; Tan et al., 2025). Currently, second-generation H1 antihistamines serve as the first-line clinical therapy; nevertheless, a substantial subset of patients suffer from insufficient therapeutic responses, high relapse rates, dose-dependent tolerability concerns, and adverse reactions such as somnolence (Chu et al., 2025; Soria, 2026). Therefore, it is urgently required to explore alternative or adjunctive therapeutic strategies with favorable safety, efficacy, and multi-target regulatory advantages.

From the perspective of traditional Chinese medicine (TCM), urticaria is primarily attributed to the invasion of wind-cold-dampness pathogenic factors into the body surface, which triggers the disharmony between ying-qi (nutrient qi) and wei-qi (defensive qi). Jingfang Mixture (JF) is derived from the classic formula “Jingfang Baidu Powder” documented in the Ming dynasty medical text *Shesheng Zhongmiao Fang*. It exerts canonical pharmacological functions of dispelling wind, eliminating cold, and removing dampness, with more than 400 years of clinical application history (Wan et al., 2021). The TCM concept that externally contracted wind-cold-dampness pathogens disturb internal physiological homeostasis shares conceptual overlaps with the modern gut-skin axis hypothesis, which proposes that intestinal microenvironmental perturbations can drive inflammatory responses in distant organs via systemic immune and metabolic signaling. Notably, pharmacological studies have revealed that pathological states corresponding to “wind-cold-dampness” are frequently accompanied by intestinal barrier and gut microbiota dysbiosis, which further echoes the gut-skin-axis theory (Zhang et al., 2024; Guo et al., 2025). Prior preclinical studies have documented the anti-inflammatory and immunomodulatory effects of JF in urticaria-related models, as well as other immune- associated disorders including autoimmune hepatitis, where it reduced inflammatory injury and cytokine dysregulation (Li et al., 2022; Zhang et al., 2023). The conceptual convergence between TCM theory and contemporary systems-biology insights inspired our hypothesis that JF ameliorates urticaria via the gut-skin axis.

When translating TCM herbal formulas into modern pharmacological evidence, appropriate disease models are indispensable for defining the interpretable and translational scope of mechanistic findings (Sun-Waterhouse et al., 2024). In the present study, we adopted the OVA/Al(OH)_3_-induced urticaria-like rat model. This model recapitulates core features of acute IgE-mediated cutaneous inflammation but cannot recapitulate the chronic, relapsing, and autoimmune trajectory observed in clinical chronic spontaneous urticaria (CSU) (He et al., 2026). Accordingly, our results should be interpreted as pre-clinical evidence for acute urticaria-like inflammation rather than directly extrapolated to CSU. To date, the molecular mechanisms underlying JF-mediated anti-urticarial effects remain incompletely elucidated. In recent years, the gut-skin axis theory has highlighted that gut microbiota and their metabolites can reshape the cutaneous immune microenvironment by modulating intestinal barrier integrity and host immune responses (Salinas et al., 2024; Zhang and Yao, 2024). Mounting evidence demonstrates that gut microbial metabolites act as pivotal functional messengers bridging commensal microbiome and host immune regulation, and participate broadly in the pathogenesis of immune-related diseases, including inflammatory bowel disease (Glassner et al., 2020), rheumatoid arthritis (Weyand and Goronzy, 2021), and urticaria (Bracken et al., 2019). Patients with urticaria frequently present gut microbial dysbiosis and metabolic disturbances, exemplified by reduced SCFA abundance and aberrant bile-acid metabolism, which may exacerbate cutaneous manifestations (Buhner et al., 2004; Cai et al., 2024; Hu et al., 2025). Collectively, we hypothesized that JF alleviates urticaria-like inflammation through a gut microbiota-metabolite-skin signaling axis, which is potentially linked to AMPK activation and suppression of p38 MAPK/PI3K-Akt suppression in the skin.

Taken together, we hypothesize that the therapeutic benefits conferred by JF are associated with reshaped gut microbial composition, altered production of key microbial metabolites, and dampened local cutaneous inflammation. Nevertheless, direct experimental evidence connecting JF-triggered alterations in the gut microbiota, metabolites, and skin immune responses is still lacking. Although the OVA/Al(OH)_3_-induced model cannot fully recapitulate the heterogeneous immune signatures and chronic relapsing clinical course of human CSU, it provides a reproducible experimental system for IgE-associated allergic inflammation that partially mirrors key immunological characteristics of urticaria. Therefore, this model was used to evaluate the systemic immunological, metabolic, and cutaneous responses upon JF intervention. To address above-mentioned knowledge gaps, we employed an ovalbumin/aluminum hydroxide OVA/Al(OH)_3_-induced rat model of urticaria-like allergic inflammation and integrated multi-omics approaches, including 16S rRNA gene sequencing, fecal untargeted metabolomics, and skin DIA-based quantitative proteomics, to systematically profile JF-driven in gut microbial composition, fecal metabolic signatures, and cutaneous protein expression patterns. Furthermore, Spearman correlation analysis was performed to explore potential associations among gut microbiota, metabolites, and skin-driven proteins. This study aims to delineate multi-omics alterations associated with JF treatment and to uncover putative gut microbiota-metabolite-skin crosstalk within this pre-clinical model, laying a foundation for follow-up mechanistic investigations.

## 2 Materials and methods

### 2.1 Drugs and reagents

Jingfang Mixture (JF, batch number: 20250329) was provided by Shandong Lunan Pharmaceutical Co., Ltd., China. According to TCM theory, JF comprises eleven medicinal herbs arranged in a clear hierarchy. *Schizonepetae Herba* (Jingjie) and *Saposhnikoviae Radix* (Fangfeng) serve as monarch drugs, synergistically dispelling wind-cold-dampness pathogens from the body surface to alleviate pruritus and promoting rash resolution. *Notopterygii Rhizoma* (Qianghuo) and *Angelicae Pubescentis Radix* (Duhuo) function as minister drugs, reinforcing wind-dispelling and damp-removing effects while alleviating pain. The adjuvant herbs encompass *Bupleuri Radix* (Chaihu) and *Peucedani Radix* (Qianhu) to regulate qi movement, *Chuanxiong Rhizoma* (Chuanxiong) to promote blood circulation and qi flow, *Aurantii Fructus* (Zhiqiao) to ameliorate middle-jiao qi stagnation, and *Poria* (Fuling) to drain dampness and invigorate the spleen; collectively, these ingredients guarantee unobstructed qi movement and prevent cold-dampness pathogens from impairing gastrointestinal function. *Platycodonis Radix* (Jiegeng) acts as the guiding herb, directing the pharmacological effects of the whole formula upward to the body surface, while *Glycyrrhizae Radix* (Gancao) harmonizes all composite herbal ingredients. Commercial enzyme-linked immunosorbent assay (ELISA) kits targeting histamine (HIS, JM-01941R1), interleukin-4 (IL-4, JM-01598R1), interleukin-6 (IL-6, JM-01597R1), interleukin-17 (IL-17, JM-01495R1), immunoglobulin E (IgE, JM-01470R1), tumor necrosis factor-α (TNF-α, JM-01587R1), diamine oxidase (DAO, JM-02209R1), and D-lactic acid (D-LAC, JM-105017R1) were purchased from Jiangsu Jingmei Biotechnology Co., Ltd. (China). HPLC-grade acetonitrile and methanol were purchased from Fisher Scientific (Fair Lawn, USA). The chemical fingerprint of JF was characterized by a Waters Acquity™ UPLC system coupled to a Waters Synapt™ G2-Si mass spectrometer. Chromatographic separation was performed on an ACQUITY UPLC™ HSS T3 column (100 mm × 2.1 mm, 1.8 µm) with acetonitrile (A) and 0.1% formic acid in water (B) as the mobile phases. The gradient elution program was set as follows: 0–2 min, 1-25% A; 2-22.5 min, 25-50% A; 22.5–24 min, 50-90% A; 24–26 min, 90-99% A; 26–28 min, 99% A (isocratic); and 28–31 min, 99-1% A. The flow rate was maintained at 0.3 mL/min, the column temperature was 40 °C, injection volume was 2 µL. Mass-spectrometric detection was operated under both positive and negative electrospray ionization modes with a mass-to-charge scan range of 50–1200 m/z. Compound identification was achieved by matching retention times, accurate molecular masses, and MS/MS fragmentation patterns against authentic reference standards and the QI database. Batch-to-batch consistency was evaluated by comparing relative peak areas of major constituents across 3 batches.

### 2.2 Experimental animals

In total, 192 healthy male rats (Sprague-Dawley, SD), weighing 180.0-200.0 g, were purchased from Liaoning Changsheng Biotechnology Co., Ltd. (License No.: SCXK (Liao) 2020-0001). All rats were housed in the Animal Safety Center of Heilongjiang University of Chinese Medicine under standard conditions (12h light/dark cycle, temperature 23 ± 2 °C, humidity 45 ± 10%) with unlimited access to sterile water and food. All animal experimental procedures complied with the *Guide for the Care and Use of Laboratory Animals*. This animal study received ethical approval from the Experimental Animal Ethics Committee of Heilongjiang University of Chinese Medicine (Ethics No.: 2025050935).

### 2.3 Animal grouping, model establishment, and drug administration

A total of 192 rats were initially assigned to 8 groups (n = 24 per group). These 24 rats per group were used for three time points (treatment day 7, 14, and 21), with n = 8 per time point. After 7 days of acclimatization, they were randomly divided into 8 groups: normal control (Con), model (Model), positive control (CH, cetirizine hydrochloride 2.0 mg/kg/day oral gavage administration), and five JF treatment groups with ascending doses (0.3, 0.6, 1.2, 2.4, 3.6 g/kg/day via intragastric gavage, designated JF1/2, JF1, JF2, JF4, JF6, respectively). Continuous intragastric administration was performed for the CH and JF groups from day 8 to day 28. The urticaria-like allergic model was constructed following previously published protocols (Peng et al., 2022). Briefly, except for the Con group, all rats received intraperitoneal injections of 0.1 mg OVA and 100 mg aluminum hydroxide on days 1, 3, and 5 for primary sensitization, followed by a booster immunization on day 12.

The clinical human dose of JF was based on a published randomized controlled trial in urticaria patients (1–2 vials, three times daily) (Zhang et al., 2025). The rat equivalent dose was calculated using the Reagan-Shaw formula based on body-surface-rea normalization: Rat dose (mg/kg) = Human dose (mg/kg) × (Km_human_/Km_rat_), where Km_rat_ = 6 and Km_human_ = 37 (Reagan-Shaw et al., 2008). The five JF doses (0.3, 0.6, 1.2, 2.4, 3.6 g/kg/day) spanned a 6-fold range centered on the calculated rat equivalent clinical dose (0.6 g/kg/day), enabling evaluation of the dose-response relationship and identification of the optimal therapeutic dosage (Li et al., 2022; Fu et al., 2026).

### 2.4 Assessment of scratching behavior

Within 30 minutes post booster immunization, scratching episodes (including forepaw scratching of the head, trunk, and biting of body tissues) of individual rats were recorded for each experimental group.

### 2.5 Histopathological analysis

Dorsal skin specimens were harvested, fixed in 4% paraformaldehyde, embedded in paraffin, sectioned (3.5 μm), and subjected to hematoxylin eosin (H&E) staining. Images were captured using a Leica Aperio CT 6 automated digital pathology scanning system. Independent scoring (0–3 for none, mild, moderate, and severe lesions) was performed by two pathologists blinded to group information based on the criteria of Peng et al (Peng et al., 2022). (0, none: no capillary dilation, no inflammatory cell infiltration; 1, mild: mild capillary dilation, mild inflammatory cell infiltration; 2, moderate: moderate capillary dilation, moderate inflammatory cell infiltration; 3, severe: severe capillary dilation, severe inflammatory cell infiltration).

Colon tissues were collected, fixed, paraffin embedded, sectioned (3.5 μm), and H&E stained. Histopathological alterations in colon segments were visualized by the Leica Aperio CT 6 scanning system.

### 2.6 Skin phenotype assessment

Dorsal skin was photographed macroscopically. Erythema, wheal-like lesions, and edema were scored. Skin thickness was measured using a digital caliper.

### 2.7 Toluidine blue staining for mast cell degranulation

Skin sections were stained with toluidine blue for 30 min. Mast cells were recognized based on metachromatic purple granules. Under 400-fold magnification, total mast cells and degranulated mast cells within three randomly selected visual fields per section were counted by two blinded investigators. The mast-cell degranulation rate was calculated as the percentage of degranulated mast cells relative to total mast cells.

### 2.8 Enzyme-linked immunosorbent assay

Serum concentrations of HIS, IL-4, IL-6, IL-17, IgE, TNF-α, DAO, and D-LAC were quantified using commercial rat-specific ELISA kits (Jiangsu Jingmei Biotechnology Co., Ltd., China) strictly following manufacturer-supplied instructions. All kits were validated by the manufacturer for rat serum sample, detailed cross-reactivity data were not provided by the manufacturer, and independent cross-reactivity testing was not performed in this study. The optical density (OD) of standards and samples were measured at 450 nm using a microplate reader. Standard curves were fitted using ELISA Calc regression. Cytokine concentrations were calculated by interpolating sample OD values against standard-curve equations. Catalog numbers, lot numbers, and detection ranges are provided in **Supplementary Table 1**.

### 2.9 Proteomics analysis

Skin tissues were ground under liquid-nitrogen. Total protein was extracted using lysis buffer supplemented with protease and phosphatase inhibitor cocktails. Protein concentrations were determined via the BCA assay. After protein reduction, alkylation, and trypsin digestion, peptides fractions were separated on an EASY-nLC 1200 nano-ultra-high-performance liquid chromatography system coupled to a Q Exactive HFX Orbitrap mass spectrometer. Separation was performed using a C18 pre-column (2cm×75μm, 3μm) and an analytical column (15cm×100μm, 1.9μm). Mobile-phase A comprised aqueous solution containing 0.1% formic acid; mobile phase B consisted of acetonitrile supplemented with 0.1% formic acid. Detailed gradient-elution and mass-spectrometry parameters are provided in **Tables 1**, **2**. Quadrupole isolation windows were adjusted according to TIMS scan time; the mass scan range covered 300-1500m/z, ion mobility ranged 0.7-1.3Vs/cm^2^, and collision energy was ramped from 20-59eV. Raw data were processed using Proteascape (BPS Version 2025b). Database searching was searched against the UniProt *Rattus norvegicus* database (UP000002494, version 20250208) concatenated with contaminant sequences. Search parameters specified trypsin cleavage specificity with maximally one missed cleavage site. Carbamidomethyl (C) was set as a fixed modification, and oxidation (M) and acetyl (protein N-term) were set as variable modifications. A target-decoy strategy was implemented; and the false discovery rate (FDR) was controlled at 0.01 for both the both peptide-spectrum match PSM and protein levels. Precursor and protein FDR were both controlled at 0.01. Proteins quantified in ≥50% of samples were retained. The analysis included (n=3) biological replicates per group. Batch effects were minimized by randomized loading order and periodic injection of quality control (QC) samples. Missing values were handled by Perseus (KNN imputation) or removed if absent in >50% of samples. Differentially expressed proteins were defined as those with *P<0.05*, |log_2_FC|≥1, and FDR<0.05.

**Table 1:** DIA Chromatographic conditions.

Time(min)	Gradient	Flow rate(μL/min)
0	7.5% B	0.7
8	35% B	0.7
9	100% B	0.7
10	100% B	0.7

**Table 2:** DIA Mass spectrum conditions.

Items	Para.
Capillary	1.6 KV
Dry Temperature	180°C
Dry Gas	3.2 L/min
Mass Range	300-1500m/z
Ion Mobility	0.7-1.3
Collision Energy	20-59eV
Ramp Time	50ms

### 2.10 16S rRNA sequencing

Fecal samples were collected and preserved at -80 °C. Total genomic DNA was extracted using commercial DNA-isolation kits following manufacturer protocols. DNA concentration and integrity were evaluated using a NanoDrop 2000 (Thermo Fisher Scientific, USA) and agarose gel electrophoresis. The V3-V4 hypervariable region of the 16S rRNA gene was amplified using the primer pair 343F (5’-TACGGRAAGGCAGCAG-3’) and 798R (5’-AGGGTATCTAATCCT-3’) using Takara Ex Taq polymerase. PCR amplicons were purified with AMPure XP magnetic beads (Agencourt), followed by a second round of PCR amplification and purification. Final amplicon libraries were quantified with the Qubit dsDNA Assay Kit (Thermo Fisher Scientific, USA) and sequenced on the Illumina NovaSeq 6000 platform with 250 bp paired-end reads (OE Biotech, Shanghai, China).

Raw sequencing outputs were stored in FASTQ format. Adapter sequences were trimmed by Cutadapt software. Read filtering, denoising, merging, and chimera removal were executed via DADA2 within QIIME2 (2020.11) with default parameters. Amplicon sequence variant (ASV) representative sequences and ASV abundance tables were generated. Taxonomic annotation for each ASV representative read was performed against the Silva database (version 138) using q2-feature-classifier with default parameters. α-Diversity (Chao1 and Shannon indices) and β-diversity (unweighted UniFrac PCoA) were computed with QIIME2 and R packages. Differential bacterial taxa were identified using ANOVA/Kruskal-Wallis/T test/Wilcoxon tests and LEfSe. Benjamini-Hochberg FDR correction was applied to LEfSe (Linear discriminant analysis effect size) results (FDR-adjusted *P<0.05*, LDA score>2) (Qian et al., 2025; Zhang et al., 2025). All samples were rarefied to 44,491 sequences (the minimum Valid-tag count) for diversity analyses; rarefaction curves reached a plateau, indicating sufficient depth.

### 2.11 Fecal untargeted metabolomics analysis

A 100 mg aliquot of fecal sample was mixed with an equal volume of methanol, vortexed, and centrifuged at 13,000r·min^-^¹ for 10min at 4 °C. Untargeted metabolomic profiling was performed on a Waters Acquity™ UPLC system coupled to a Waters Synapt™ G2-Si mass spectrometer. Chromatographic separation for fecal samples was achieved on a Waters ACQUITY UPLC HSS T3 column (100mm×2.1mm, 1.8μm) maintained at 35 °C with a flow rate of 0.3 mL/min. Mobile-phase consisted of water containing 0.1% formic acid (A) and acetonitrile containing 0.1% formic acid (B), with the optimized gradient elution program as follows: 0.0-1.0min, 1%-78% B; 1.0-4.0min, 78%-84% B; 4.0-9.0min, 84%-99% B; 9.0-10.0min, 99% B; 10.0-11.0min, 1% B. Mass spectrometry analysis was performed in both positive (ESI^+^) and negative (ESI^-^) electrospray ionization modes. Source parameters were set as follows: capillary voltage 2.8 kV, source temperature 110 °C, cone voltage 40 V, cone gas flow 50L/h, desolvation gas flow 800L/h, and desolvation temperature 400 °C. Data were acquired in centroid mode with a mass range of m/z 50-1200Da.

Raw LC-MS data were pre-processed by Compound Discoverer software for noise reduction, baseline correction, and peak alignment across all sample datasets. QC samples, prepared by pooling equal aliquots from all experimental specimens, were injected intermittently to monitor technical variability. Metabolic features with relative standard deviation (RSD)>30% in QC samples were excluded from subsequent analysis. Metabolite annotation was accomplished by matching accurate mass (<5ppm error) and MS/MS fragmentation spectra (when available) against the Human Metabolome Database (HMDB), METLIN database, and an in-house library of authenticated reference standards. Metabolite identification confidence levels were assigned according to guidelines from the Metabolomics Standards Initiative (MSI).

### 2.12 Immunohistochemistry

Skin paraffin sections (3.5μm) were deparaffinized, rehydrated, and antigen-retrieved in citrate buffer (pH 6.0). After blocking non-specific binding sites with 5% bovine serum albumin (BSA), sections were incubated overnight at 4 °C with primary antibodies targeting p-AMPK (Affinity, AF3423; 1:100), p-p38 MAPK (Santa, sc-7973; 1:50), p-Akt (Proteintech, 66444-1-Ig; 1:200), and p-PI3K (Affinity, AF3242; 1:100). After repeated washing steps, sections were incubated with HRP (horseradish peroxidase) -conjugated secondary antibodies (Cell Signaling Technology (CST), 7074 for rabbit, 7076 for mouse; 1:500), followed by 3,3′ diaminobenzidine (DAB) detection and hematoxylin counterstaining. Under 400-fold magnification, integrated optical density (IOD) from three randomly selected visual fields per section was quantified with ImageJ software by two investigators blinded to experimental grouping.

### 2.13 Western blot

Skin tissues were lysed in radioimmunoprecipitation assay (RIPA) lysis buffer containing protease/phosphatase-inhibitors. Proteins (30μg) were separated by sulfate polyacrylamide gel electrophoresis (SDS PAGE) and electro-transferred onto polyvinylidene fluoride (PVDF) membranes. After blocking with 5% BSA, membranes were incubated overnight at 4 °C with primary antibodies against phosphorylated proteins: p-AMPK(Thr172) (Proteintech, 80209-6-RR; 1:1000), p-p38(Thr180/Tyr182) (Proteintech, 28796-1-AP; 1:1000), p-PI3K(Tyr458)/p55 (Tyr199) (CST, 4228; 1:1000), and p-Akt (Ser473) (CST, 4060; 1:1000); and total proteins: AMPKα (Proteintech, #10929-2-AP; 1:1000), p38 MAPK (Proteintech, 14064-1-AP; 1:1000), PI3K (Proteintech, 20584-1-AP; 1:1000), and Akt (CST, 9272; 1:1000). β-actin (Proteintech, 66009-1-lg; 1:1000) was used as loading control. Membranes were subsequently incubated with HRP-conjugated goat anti-rabbit secondary antibody (Proteintech, RGAR001; 1:5000). Protein bands were visualized by enhanced chemiluminescence (ECL). Band intensity quantification was performed using ImageJ software. Phosphorylated-protein abundances were normalized to the corresponding total-protein levels.

### 2.14 Statistical analysis

All quantitative data are presented as mean ± standard deviation (SD). Prior to parametric statistical testing, normality was validated by the Shapiro-Wilk test, and homogeneity of variance was assessed using Levene’s test. When both prerequisites were satisfied, one-way analysis of variance (ANOVA) followed by Tukey’s *post-hoc* test was applied for multiple-group comparisons. When normality or homogeneity of variance assumptions were violated, the Kruskal-Wallis test coupled with Dunn’s *post-hoc* test was adopted. For metabolomics, differential metabolites were screened based on orthogonal partial least squares discriminant analysis (OPLS DA) with variable importance in projection (VIP) >1.0, combined with *P<0.05* and |log_2_FC|≥1. For proteomics, DEPs were defined with criteria *P < 0.05*, |log_2_FC|≥1, and Benjamini-Hochberg FDR<0.05. For Spearman correlation analysis and LEfSe, Benjamini-Hochberg FDR correction was applied (FDR-adjusted *P<0.05*). Statistically significant difference was defined when *P < 0.05.* For 16sRNA, LEfSe analysis was performed with LDA score>2, and Benjamini-Hochberg FDR correction was applied (FDR-adjusted *P<0.05*). The list of abbreviations is in **Supplementary Table 3**.

## 3 Results

### 3.1 Chemical characterization of JF

The UPLC-MS fingerprint of JF is illustrated in **Supplementary Figure 1**. In total, 152 major chemical constituents were tentatively annotated, including Prim-O-glucosylcimifugin, 5-O-methylvisammioside, Saikosaponin A, Saikosaponin D, glycyrrhizic acid, liquiritin, and ferulic acid (**Supplementary Table 2**). Among these compounds, terpenoids and flavonoids have been previously reported to modulate AMPK signaling cascades and suppress inflammatory responses (Hao et al., 2024), implying that these bioactive ingredients may contribute to JF-derived therapeutic efficacy observed in this study. The RSD values for major characteristic constituents across 3 batches were below 5%, demonstrating satisfactory batch-to-batch consistency.

### 3.2 JF alleviates cutaneous inflammation and pruritus in a urticaria rat model

To evaluate the *in-vivo* protective efficacy of JF, the OVA/Al(OH)_3_-induced urticaria rat model was constructed. Model-group rats exhibited frequent scratching and loose stools, indicating successful model establishment. JF intervention mitigated these pathological manifestations in a dose- and time-dependent manner. H&E staining revealed prominent skin lesions in the model group including epidermal hyperplasia, dermal edema, and robust inflammatory cell infiltration. All JF-treated groups displayed varying degrees of histological improvement, with the JF 2, JF 4, and JF 6 groups exerted exhibiting superior protective potency (**Figures 1A, B**).

**Figure 1:**
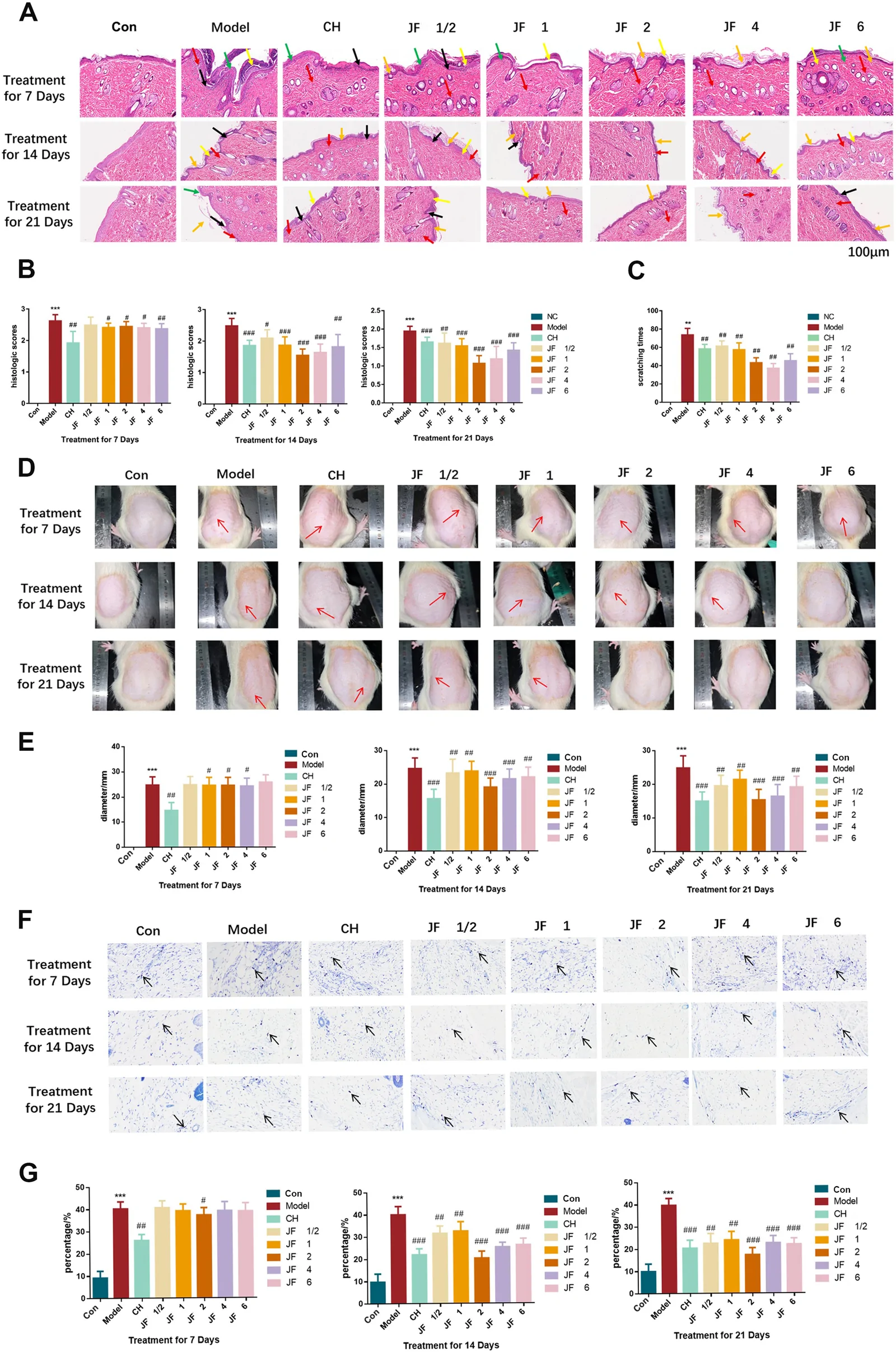
Protective effect of JF on OVA/aluminium hydroxide-induced urticaria-like skin inflammation in rats. **(A)** Representative H&E-stained skin sections. Black arrow, necrosis/epidermal loss; yellow arrow, abscess; green arrow, incomplete keratinization; orange arrow, hyperkeratosis; red arrow, inflammatory infiltration. Scale bar=100µm. **(B)** Histologic scores. **(C)** Scratch number. **(D)** Gross skin images. **(E)** Skin thickness. **(F)** Toluidine blue staining of mast cells; arrows indicate degranulated mast cells. Scale bar=50 µm. **(G)** Mast cell degranulation rate. Data are mean ± SD (n=8). One-way ANOVA with Tukey’s/Dunnett’s *post hoc* test after normality/variance tests (Kruskal-Wallis with Dunn’s test for non-normal data); Exact P values in **Supplementary Table 5**. **P<0.01, ***P<0.001 vs. Con; ##*P<0.01*, ###*P<0.001* vs. Model.

At treatment day 14, these three JF groups achieved better therapeutic outcomes compared with the positive control (CH group); by treatment day 21, all JF- intervention groups outperformed the CH group, indicating obvious time-cumulative pharmacological effects. Scratching-behavior assays further validated that JF significantly reduced scratching frequency and ameliorated OVA/Al(OH)_3_-provoked skin injury (**Figure 1C**). Collectively, JF exerted prominent protective effects against urticaria-like lesions in rats. Among all JF-treated groups, the JF2 group achieved the most pronounced therapeutic improvement at day 21. Compared with the Con group, Model rats exhibited apparent erythema, wheal-like lesions, and increased skin thickness (*P<0.01*). JF2 treatment significantly reversed these skin manifestations (*P<0.01*) (**Figures 1D, E**). Toluidine-blue staining showed that the mast-cell degranulation ratio was drastically elevated in the Model group versus the Con group (*P<0.001*), whereas JF2 significantly reduced mast-cell degranulation (*P<0.01*) (**Figures 1F, G**). Comparative effect-size analyses revealed that JF2 exhibited superior efficacy relative JF6 in terms of erythema area (Hedges’ g=-1.172, 95% CI: -2.179 to -0.165; adjusted P = 0.038), mast-cell counts (Hedges’ g=-1.503, 95% CI: -2.561 to -0.445; adjusted P = 0.011), and histological score (Hedges’ g=-1.758, 95% CI: -2.896 to -0.620; adjusted P = 0.010). JF2 also outperformed JF4 in terms of mast-cell abundance (Hedges’ g=-1.607, 95% CI: -2.683 to -0.531; adjusted P = 0.010). For erythema area of skin, JF2 was lower than JF4, but the difference was not statistically significant (Hedges’ g=-0.319, 95% CI: -1.249 to 0.611; adjusted P = 0.612). Although JF2 yielded lower than JF 6 but the difference was not statistically significant (Hedges’ g=-0.281, 95% CI: -1.894 to 1.332; adjusted P = 0.690) (**Supplementary Table 4**). Therefore, the JF2 group (1.2g/kg/day, approximately twice the clinical equivalent rat dose) was selected for subsequent mechanistic investigations based on its optimal therapeutic performance.

### 3.3 JF suppresses the systemic allergic-inflammatory response and improves intestinal barrier markers

To characterize JF’s impacts on systemic inflammation and intestinal-barrier integrity, serum immunoglobulin levels, inflammatory mediators, intestinal-permeability biomarkers, as well as colonic histopathology were examined. Compared with the Con group, the model group displayed significantly elevated serum IgE and IL-4 (*P<0.01*) and histamine (*P<0.001*), indicative of Th2-skewed immune activation and mast-cell degranulation. Serum IL-6, TNF-α, and IL-17 concentrations were also markedly increased (*P<0.01*), demonstrating robust systemic inflammatory activation. JF reduced all of these markers (*P<0.01*) (**Figure 2A**); the reduction in IL-6 and TNF-α was accompanied by alleviated skin inflammatory cell-infiltration (**Figure 1**).

**Figure 2:**
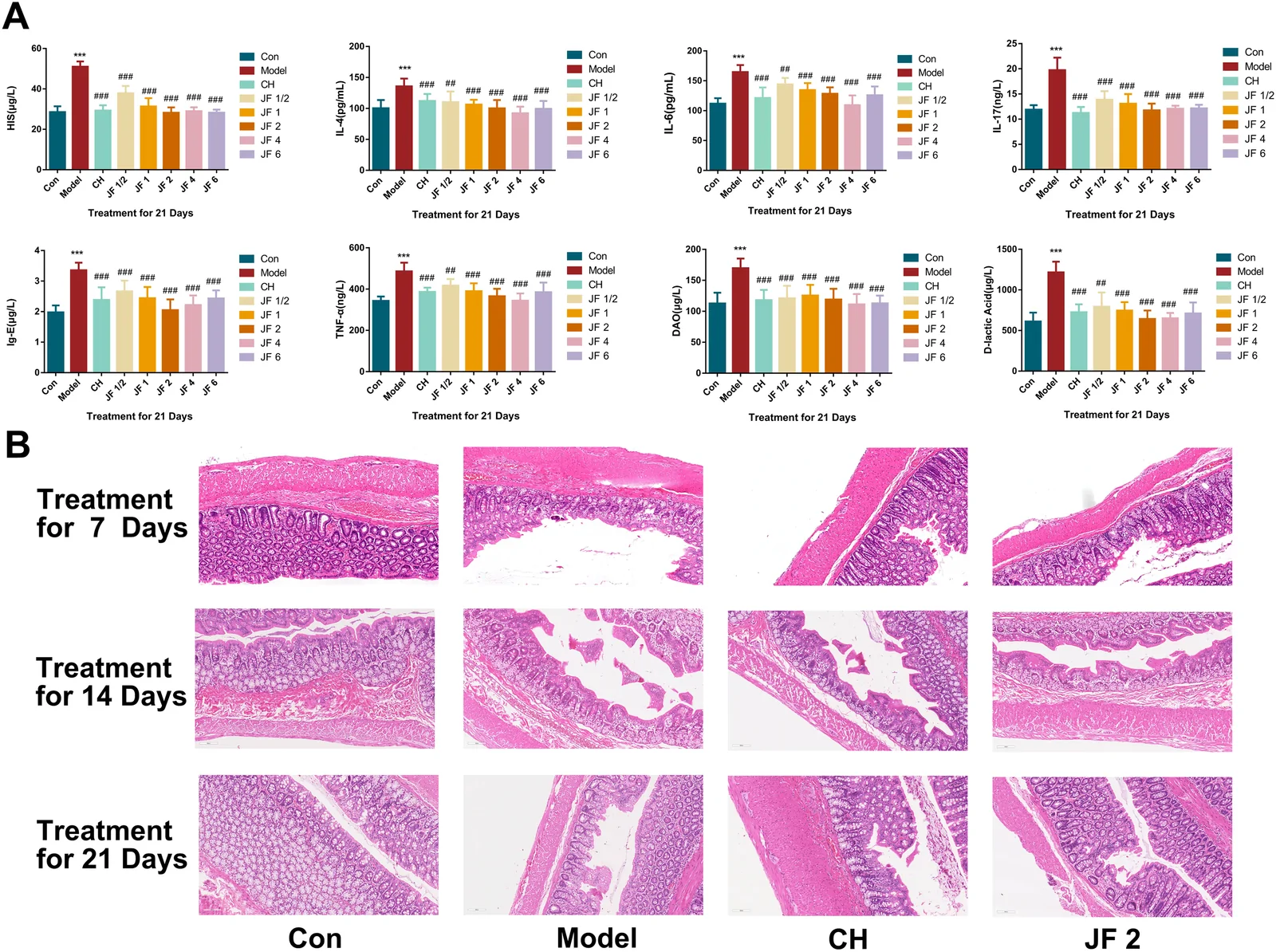
Effects of JF on inflammation and intestinal barrier in OVA/aluminium hydroxide-induced urticaria-like skin inflammation rats. **(A)** Serum histamine (HIS), IL-4, IL-6, IL-17, IgE, TNF-α, DAO, and D-LAC (n=8). **(B)** Representative H&E-stained colon sections; scale bar=100µm. (n=3). One-way ANOVA with Tukey’s/Dunnett’s *post hoc* test after normality/variance tests (Kruskal-Wallis with Dunn’s test for non-normal data); Exact P values in **Supplementary Table 5**. **P<0.01, ***P<0.001 vs. Con; ##*P<0.01*, ###*P<0.001* vs. Model.

Tight junctions (TJs) protein complexes are indispensable for maintaining intestinal physiological homeostasis and defending the host against luminal harmful substances (Otani and Furuse, 2020). Serum DAO and D-LAC serve as reliable circulating biomarkers reflecting intestinal-barrier structural integrity and altered intestinal permeability (Zhuang et al., 2019). Regarding intestinal-barrier function, DAO and D-LAC concentrations were drastically augmented in the model group, JF administration effectively reversed these aberrant elevations (*P < 0.05*) (**Figure 2A**). Colonic H&E staining revealed epithelial alterations and inflammatory-cell infiltration in the model group, whereas JF-treated rats exhibited restored colonic mucosal architecture (**Figure 2B**). Among the treatment groups, JF2 gave the greatest improvement in both permeability markers and mucosal structure. JF significantly reduced serum DAO and D-LAC levels and improved colonic mucosal architecture, suggesting an improvement in intestinal barrier-associated abnormalities. Notably, direct detection of tight-junction proteins, *in-situ* intestinal-permeability assays, and circulating endotoxin/LPS quantification were not performed in this study (see Study Limitations).

### 3.4 JF reverses gut microbiota dysbiosis in urticaria model rats by reducing *Proteobacteria* and restoring SCFA-producing bacteria

16S rRNA gene-sequencing analysis was performed to dissect JF-mediated modulatory effects on gut microbial communities. α-diversity (Chao1 and Observed species indices) were significantly reduced in the model group compared with the Con group (*P<0.05*), and were partially restored following JF intervention (**Figures 3A, B**). β-diversity analysis revealed clear separation of gut microbial community composition among the Con, model, and JF groups (**Figures 3C, D**). At the phylum level, the relative abundance of *Proteobacteria* was increased in the model group, while *Bacteroidetes* was decreased. At the order level, *Lachnospirales* and *Oscillospirales* were decreased. JF reversed these microbial compositional perturbations (**Figures 3E–G**). LEfSe analysis identified discriminatory bacterial taxa across multiple taxonomic levels: at the order level, *Enterobacterales* was enriched in the model group; at the family level, *Enterococcaceae* was enriched in the model group; at the genus level, *Bacteroides* and *Lachnospira* were enriched in the JF group (**Figures 3H, I**).

**Figure 3:**
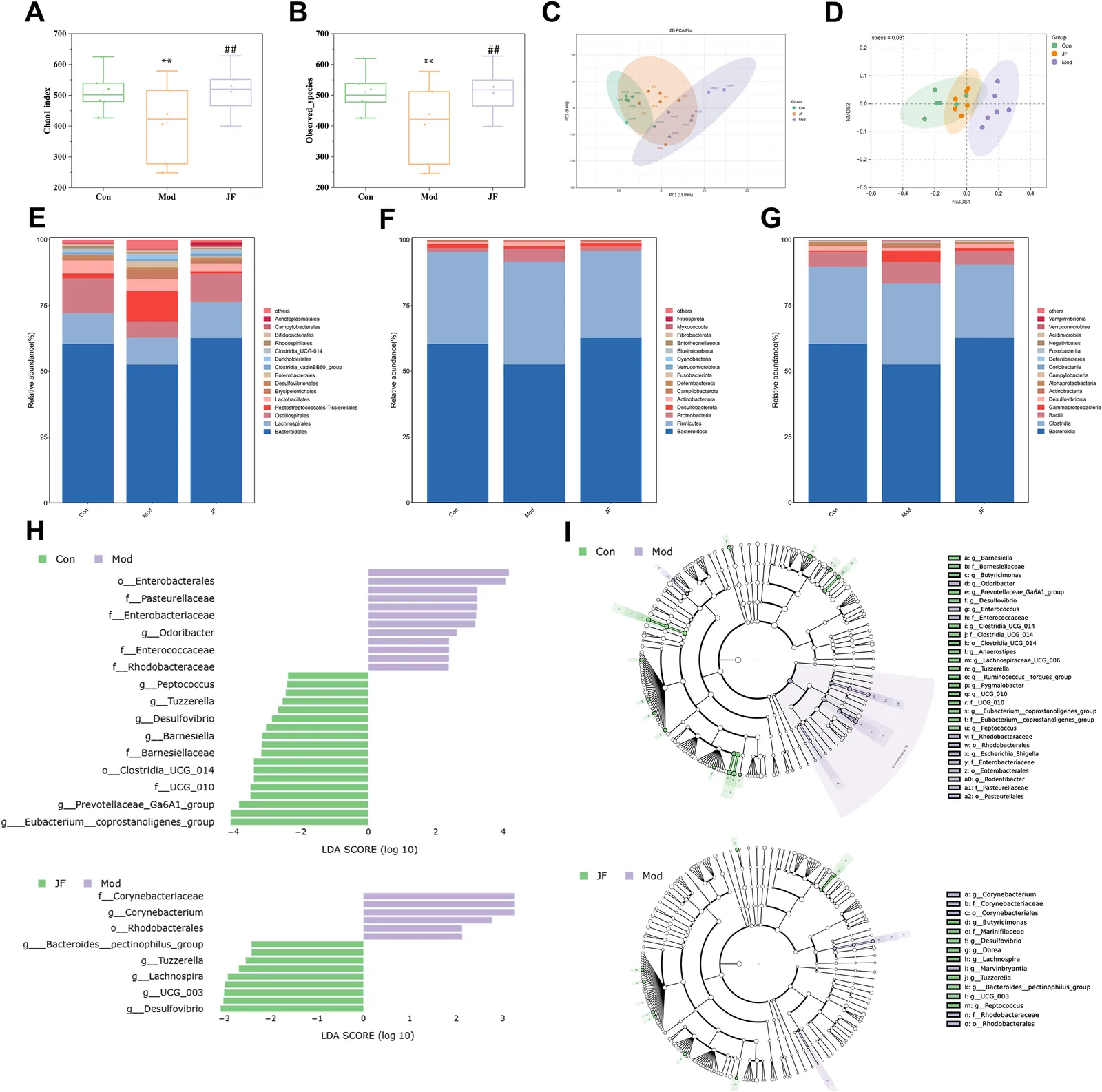
Effect of JF on gut microbiota diversity in OVA/aluminium hydroxide-induced urticaria-like skin inflammation rats. **(A)** Chao1 index. **(B)** Observed species index. **(C)** PCA score plot. **(D)** Non-metric multidimensional scaling (NMDS) plot (Stress=0.031). **(E–G)** Relative abundance at phylum, class, and order levels. **(H)** LEfSe cladogram. **(I)** LDA score plots (LDA>2). Taxonomic levels: p, phylum; c, class; o, order; f, family; g, genus; s, species. Bacterial names are italicized. Data are mean ± SD (n=6). Alpha diversity: one-way ANOVA/Kruskal-Wallis; beta diversity: PERMANOVA; LEfSe: LDA>2 with FDR correction. Exact P values in **Supplementary Table 5**. **P<0.01 vs. Con; ##P<0.01 vs. Model.

The heatmap analysis of differentially abundant taxa across taxonomic levels was consistent with these findings (**Figure 4A**). In the model group, at the phylum level, *Proteobacteria* was elevated; at the order level, *Enterobacterales* was elevated; and at the family level, *Enterococcaceae* was elevated. These microbial groups are well-documented to drive intestinal-barrier disruption and propagate inflammatory cascades (Janda and Abbott, 2021; Zigmond et al., 2023; Zhang et al., 2025). Meanwhile, multiple beneficial taxa were depleted. At the class level, *Bacteroidia* and *Clostridia* were depleted. At the order level, *Bifidobacteriales*, *Erysipelotrichales*, *Oscillospirales*, and *Lachnospirales* were depleted. At the family level, *Barnesiellaceae* and the *Coprostanoligenes* group were depleted. At the genus level, *Lachnospira*, *Odoribacter*, *Butyricimonas*, and *Anaerostipes* were depleted. At the species level, *Lactobacillus intestinalis* and *Parabacteroides distasonis* were depleted. Most of these taxa contribute to short-chain fatty acid (SCFA) production, barrier maintenance, and immune regulation (Zhang et al., 2026). JF treatment largely reversed these microbial compositional derangements (**Figures 4B–F**).

**Figure 4:**
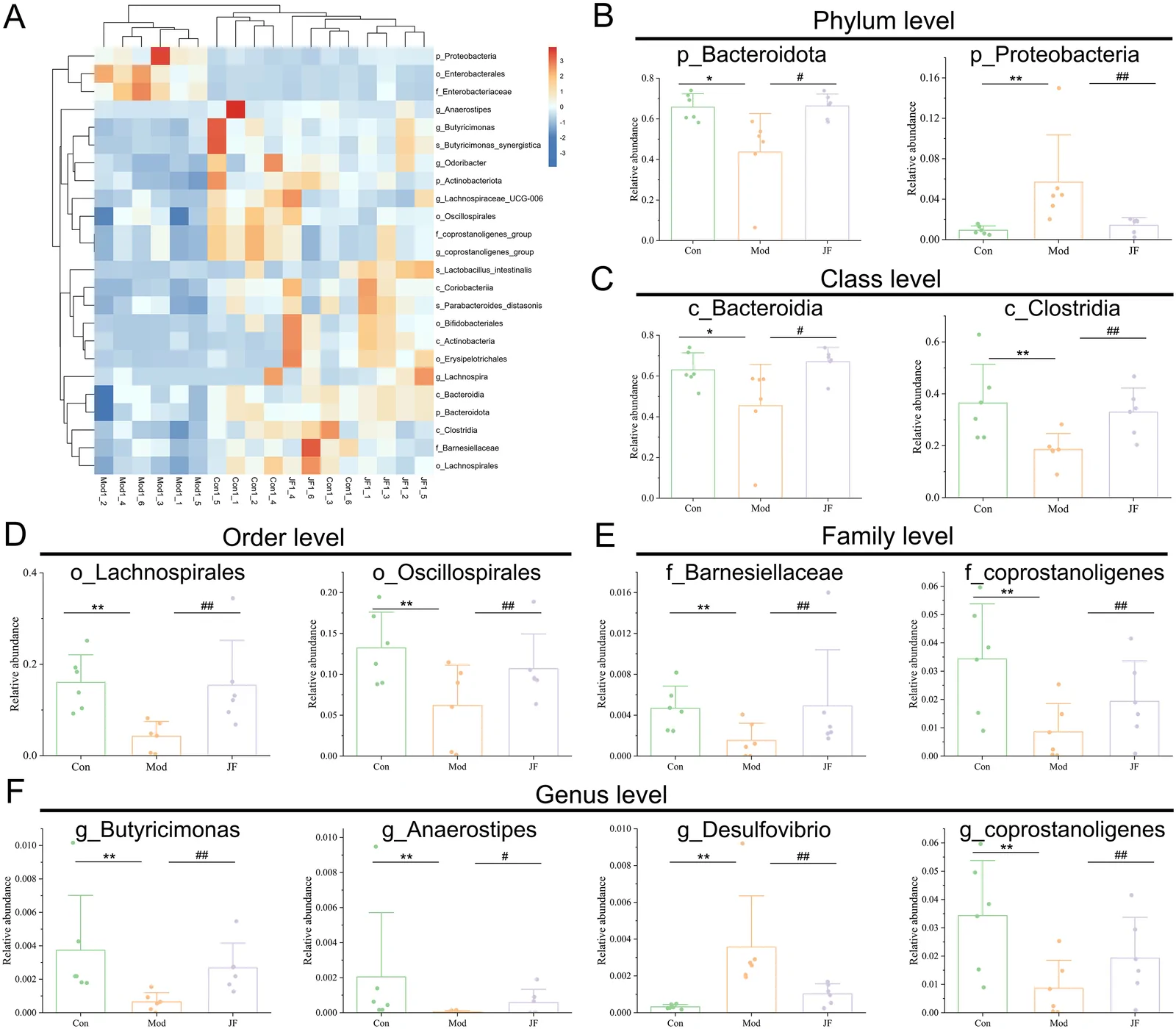
Effect of JF on gut microbiota disturbance. **(A)** Heatmap of differential microbiota at phylum, class, order, family, and genus levels; red, high abundance; blue, low abundance. Taxonomic levels are clearly labeled. **(B–F)** Abundance of differential bacteria at phylum, class, order, family, and genus levels. Data are mean ± SD (n=6). LEfSe: LDA>2 with FDR correction; between-group comparisons by one-way ANOVA/Kruskal-Wallis with *post hoc* correction. Exact P values in **Supplementary Table 5**. *P<0.05, **P<0.01, ***P<0.001 vs. Con; ##*P<0.01*, ###*P<0.001* vs. Model.

### 3.5 JF normalizes the fecal metabolome, raising SCFA and tryptophan metabolites and lowering pro-inflammatory bile acids and LTB4

Gut microbiota most of their biological functions via bioactive microbial metabolites (Wang et al., 2024). Accordingly, untargeted LC-MS/MS-based metabolomics was applied to characterize fecal metabolites. PCA separated the Con, model, and JF groups (**Figure 5A**), indicating that urticaria-like inflammation remodeled the fecal metabolome, while JF treatment shifted metabolic profiles back toward the normal state of the Con group. OPLS-DA combined with VIP-based screening, identified 24 differential metabolites (**Table 3**). Relative to the Con group, the model group exhibited significantly reduced fecal levels of butyric acid, indole, indole-3-acetic acid, and docosahexaenoic acid (DHA) (*P<0.05*). These metabolites exert well-established anti-inflammatory and immunomodulatory activities. Meanwhile, pro-inflammatory mediators including deoxycholic acid, chenodeoxycholic acid, arachidonic acid (AA), and leukotriene B4 (LTB4) were significantly accumulated in the Model group (*P<0.05*). which are known pro-inflammatory mediators. Metabolic-pathway enrichment analysis indicated that these differentially metabolites were primarily involved in short-chain fatty acid (SCFA) metabolism, bile acid metabolism, tryptophan metabolism, and lipid metabolism (**Figure 5B**). JF intervention significantly restored all these metabolic abnormalities. (**Figure 5C**). Representative differential metabolites are visualized in **Figures 5D–G**.

**Figure 5:**
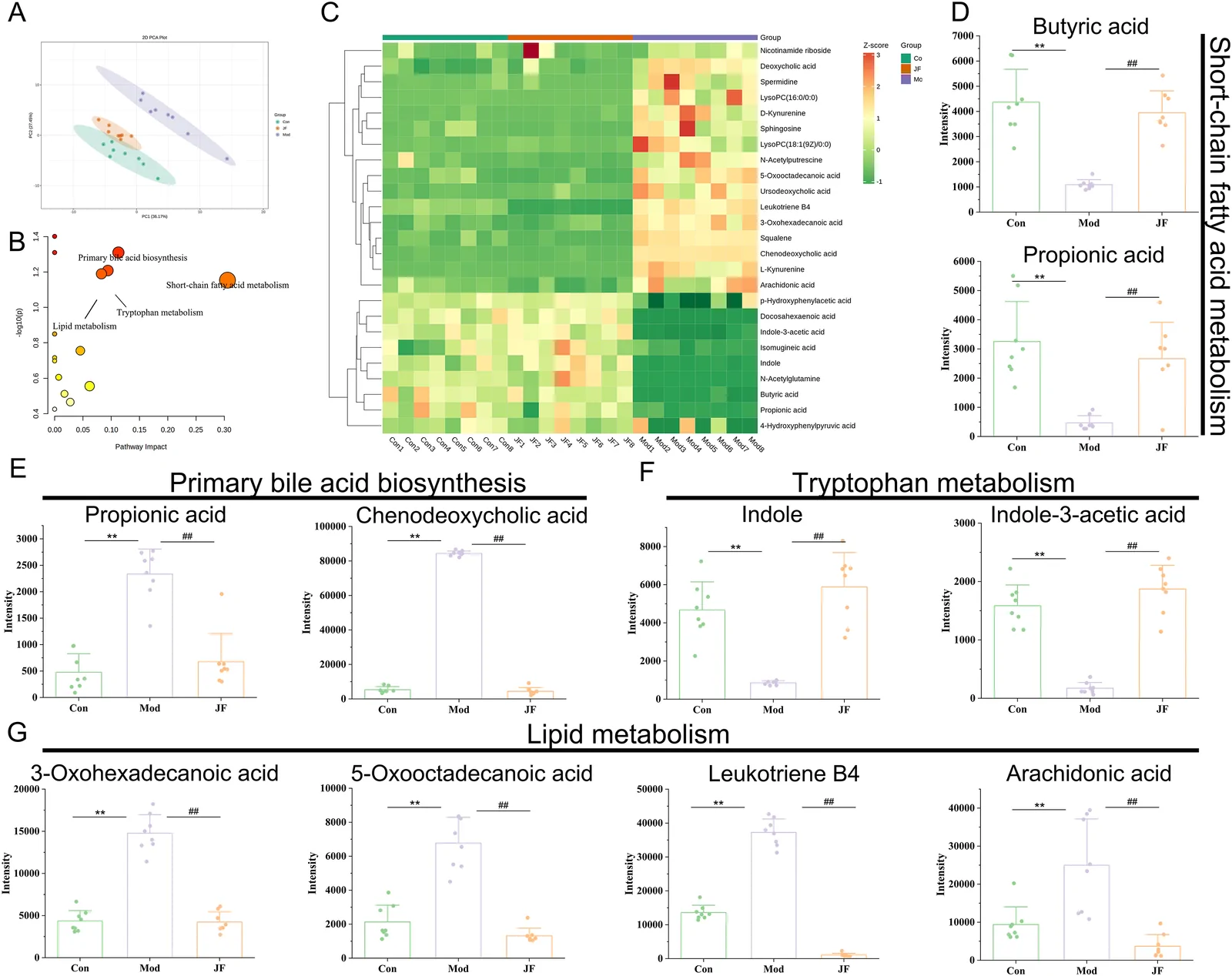
Effect of JF on fecal metabolites in OVA/aluminium hydroxide-induced urticaria-like skin inflammation rats. **(A)** PCA score plot. **(B)** Pathway enrichment analysis showing main enriched pathways (e.g., SCFA, bile acid metabolism, tryptophan metabolism, lipid metabolism). **(C)** Heatmap of differential metabolites (red: high, green: low). **(D–G)** SCFA, bile acid, tryptophan, and lipid metabolites. Data are mean ± SD (n=8). Differential metabolites: VIP>1 and FDR<0.05; pathway enrichment: hypergeometric test with FDR correction; between-group comparisons by one-way ANOVA/Kruskal-Wallis with *post hoc* correction. Exact P values in **Supplementary Table 5**. **P<0.01 vs. Con; ##P<0.01 vs. Model.

**Table 3:** Identification of potential feces biomarkers in urticaria model rats.

No.	T R/min	Metabolites	Molecular formula	Relative molecular mass	Ion mode	HMDB	Change trend	
							Mod vs NC	Mod vs JFM
1	2.70	Butyric acid	C_4_H_8_O_2_	89.0597	ESI^+^	HMDB0000039	↓	↑
2	0.79	Propionic acid	C_3_H_6_O_2_	149.0805	ESI^+^	HMDB0000237	↓	↓
3	6.50	Deoxycholic acid	C_24_H_40_O_4_	393.2998z	ESI^+^	HMDB0000626	↑	↓
4	5.17_	Chenodeoxycholic acid	C_24_H_40_O_4_	415.2831	ESI^+^	HMDB0000518	↑	↓
5	3.78	Indole	C_8_H_7_N	235.1221	ESI^+^	HMDB0000738	↓	↑
6	2.31	Indole-3-acetic acid	C_12_H_13_NO_2_	226.0835	ESI^+^	HMDB0304946	↓	↑
7	1.02	L-Kynurenine	C_10_H_12_N_2_O_3_	207.0775	ESI^+^	HMDB0000684	↑	↓
8	3.38	D-Kynurenine	C_10_H_12_N_2_O_3_	417.1782	ESI^+^	HMDB0250769	↑	↓
9	0.87	N-Acetylglutamine	C_7_H_12_N_2_O_4_	187.0725	ESI^+^	HMDB0006029	—	—
10	0.86	N-Acetylputrescine	C_6_H_14_N_2_O	129.1033	ESI^+^	HMDB0002064	↑	↓
11	9.82	3-Oxohexadecanoic acid	C_16_H_30_O_3_	269.2122	ESI^+^	HMDB0010733	↑	↓
12	4.45	5-Oxooctadecanoic acid	C_18_H_34_O_3_	343.2489	ESI^+^	HMDB0034074	↑	↓
13	9.53	Squalene	C_30_H_50_	821.7928	ESI^+^	HMDB0000256	↑	↓
14	5.15	Ursodeoxycholic acid	C_24_H_40_O_4_	392.2926	ESI^+^	HMDB0000946	↑	↓
15	0.75	Spermidine	C_7_H_19_N_3_	146.1650	ESI^+^	HMDB0001257	↑	↓
16	0.81	Nicotinamide riboside	C_11_H_15_N_2_O_5_	278.0868	ESI^+^	HMDB0000855	—	—
17	7.11	Sphingosine	C_18_H_37_NO_2_	344.2806	ESI^+^	HMDB0000252	↑	↓
18	4.18	Docosahexaenoic acid	C_22_H_32_O_2_	373.2388	ESI^+^	HMDB0002183	↓	↑
19	3.47	Arachidonic acid	C_20_H_32_O_2_	349.2383	ESI^+^	HMDB0001043	↑	↓
20	6.85	LysoPC(18:1(9Z)/0:0)	C_26_H_52_NO_7_P	566.3462	ESI^-^	HMDB0002815	↑	↓
21	6.70	LysoPC(16:0/0:0)	C_24_H_50_NO_7_P	540.3308	ESI^-^	HMDB0010382	↑	↓
22	4.17	Leukotriene B4	C_20_H_32_O_4_	335.2227	ESI^-^	HMDB0001085	↑	↓
23	0.84	Isomugineic acid	C_12_H_20_N_2_O_8_	365.1205	ESI^-^	HMDB0033555	↑	↓
24	2.60	4-Hydroxyphenylpyruvic acid	C_9_H_8_O_4_	359.0774	ESI^-^	HMDB0000707	↑	↓

Notably, butyric acid and indole derivatives are gut-microbiota-derived bioactive molecules with potent anti-inflammatory and immunoregulatory capacities. Conversely, deoxycholic acid, chenodeoxycholic acid, arachidonic acid, and its downstream metabolite LTB4 represent classical pro-inflammatory mediators. These metabolic shifts were tightly correlated with JF-triggered gut-microbiota remodeling (Huang et al., 2021; Zhang et al., 2021). Collectively, these results suggest that JF effectively reverses urticaria-associated fecal metabolic dysregulation by restoring SCFA, tryptophan, and lipid metabolism. It elevates anti-inflammatory metabolites (butyrate, indole derivatives, DHA) while reducing pro-inflammatory bile acids and LTB4, thereby rebalancing the intestinal metabolic microenvironment.

### 3.6 JF may be associated with restoration of skin AMPK signaling while suppressing aberrant p38 MAPK and PI3K/Akt signaling pathway activation in urticaria model rats

To elucidate the molecular mechanisms underlying JF’s anti-urticarial efficacy, DIA-based quantitative proteomics was employed to analyze protein expression profiles in skin tissues. Under screening thresholds *P<0.05* and |log_2_FC|≥1, volcano plot analysis (**Figures 6A, B**) identified 107 differentially expressed proteins (53 upregulated, 54 downregulated) between the model and control groups, and 243 differentially expressed proteins (128 upregulated, 115 downregulated) between the JF and model groups. The markedly expanded number of DEPs upon JF treatment suggests that JF triggers broad-spectrum remodeling of the cutaneous protein regulatory network.

**Figure 6:**
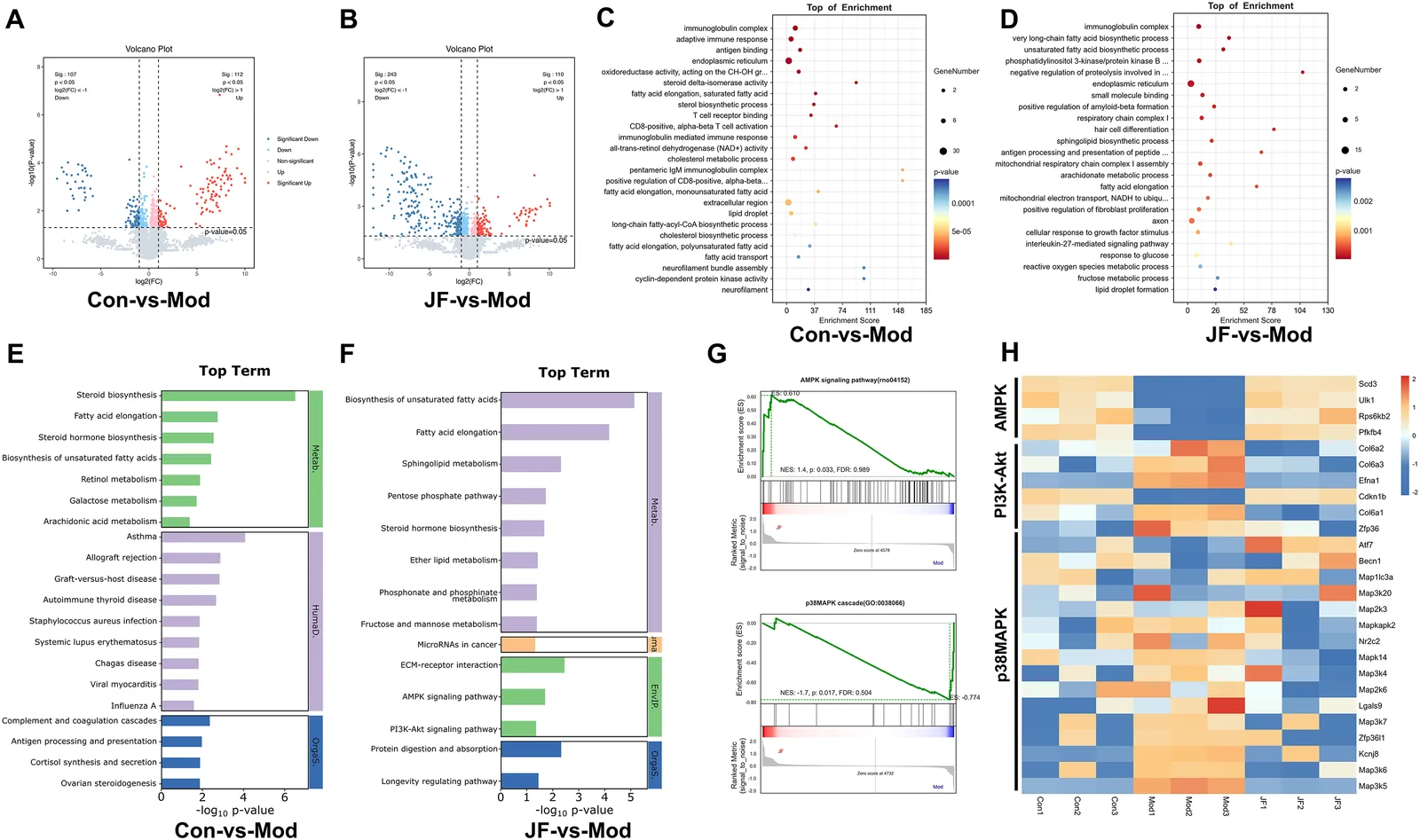
Effect of JF on skin protein expression in OVA/aluminium hydroxide-induced urticaria-like skin inflammation rats. **(A)** Volcano plot, Model vs. Con. **(B)** Volcano plot, JF vs. Model. Red, upregulated; blue, downregulated; gray: non-significant proteins; dashed lines: FDR = 0.05 and |log_2_FC|=1. **(C)** GO enrichment (BP, CC, MF). **(D)** Extended GO enrichment. **(E)** KEGG enrichment, Model vs. Con. **(F)** KEGG enrichment, JF vs. Model. **(G)** AMPK pathway enrichment score. **(H)** Heatmap of AMPK/p38 MAPK/PI3K-Akt-related proteins. Data are mean ± SD (proteomics n=3 per group). Differential proteins: FDR<0.05 and |log_2_FC|≥1; GO/KEGG: Fisher’s exact test with FDR correction.

GO (Gene Ontology) functional enrichment analysis (**Figures 6C, D**) revealed that DEPs between the model and control groups were mainly enriched in immune-related functions, including immunoglobulin complex, adaptive immune response, antigen binding, T cell receptor binding, and CD8^+^ T cell activation, and lipid metabolic processes such as fatty acid elongation, steroid biosynthesis, and cholesterol metabolism. In contrast, DEPs obtained from JF versus model groups were significantly enriched in mitochondrial respiratory-chain complex I, complex I assembly, and mitochondrial electron transport-a pivotal finding. Complex I serves as the rate-limiting component for oxidative phosphorylation, directly governing ATP production and intracellular NAD^+^/NADH ratio, it serves as a critical upstream modulator of AMPK activity (Hou et al., 2018; Falk et al., 2026).

Kyoto Encyclopedia of Genes and Genomes (KEGG) pathway enrichment analysis further revealed (**Figures 6E, F**) that DEPs in the model group were enriched in lipid metabolism pathways (steroid biosynthesis, fatty-acid elongation, unsaturated-fatty acid-biosynthesis, and arachidonic-acid metabolism) as well as multiple immune-related signaling cascades. For the JF-intervention group, DPEs were significantly enriched in lipid-metabolism (unsaturated-fatty-acid biosynthesis, fatty-acid elongation, sphingolipid metabolism, and ether-lipid metabolism), along with the AMPK and PI3K-Akt signaling pathways. Gene-set-enrichment analysis (GSEA) independently validated significant enrichment of the AMPK signaling pathway in the JF group, whereas the p38 MAPK pathway was enriched in the model group (**Figure 6G**). AMPK serves as a central hub integrating cellular energy metabolism and inflammatory regulation; its activation suppresses pro-inflammatory responses and modulates lipid metabolism (Hardie, 2004; O'Neill and Hardie, 2013). The enrichment of the p38 MAPK pathway in the model group suggests its involvement in urticaria-associated inflammation. Collectively, these multi-omics data indicate that AMPK signaling is suppressed while the p38 MAPK cascades are pathologically over-activated in the model group, and JF treatment reverses this pathological signaling status. Heatmap visualization profiling protein abundance for core components of the AMPK-p38 MAPK-PI3K/Akt axis further illustrated JF-driven alterations in these signaling molecules (**Figure 6H**).

### 3.7 Spearman correlation analysis suggests potential associations among gut microbiota, metabolites, and skin proteins

To establish potential links between JF-responsive gut microbiota, fecal metabolites, and skin proteins, we performed Spearman correlation analysis incorporating differential bacterial taxa across taxonomic ranks, differential fecal metabolites, and key proteins belonging to the skin AMPK/p38 MAPK/PI3K-Akt signaling axis identified via proteomics. The results are presented as correlation heatmaps (**Figure 7**).

**Figure 7:**
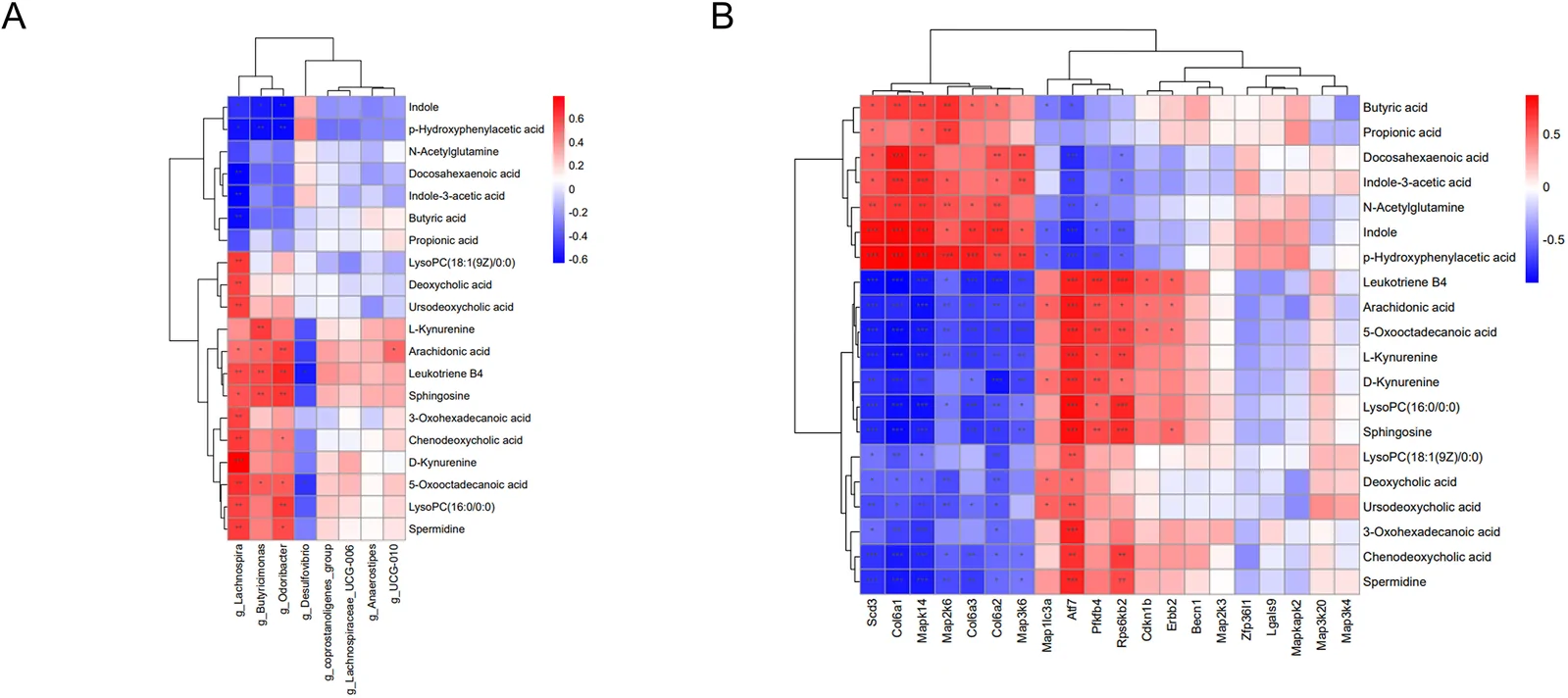
Exploratory correlation analysis and proposed mechanism. **(A)** Spearman correlations between differential gut microbiota at the genus level and fecal differential metabolites. **(B)** Spearman correlations between differential fecal metabolites and AMPK/fatty acid metabolism-related skin proteins. Red: positive correlation; blue: negative correlation. Bacterial names are italicized. Data are from n=3 per group. (FDR-adjusted P). *P<0.05, **P<0.01, ***P<0.001. Exact P values in **Supplementary Table 5**. These analyses are exploratory and hypothesis-generating, not causal.

Regarding microbiota-metabolite correlations (**Figure 7A**), at the genus taxonomic level, *Lachnospira*, *Butyricimonas*, *Odoribacter*, and *Anaerostipes* exhibited significant correlations with anti-inflammatory metabolites (arachidonic acid, 3-oxohexadecanoic acid, 5-oxooctadecanoic acid, deoxycholic acid, etc.) (*P<0.05*), whereas genus *Desulfovibrio* was negatively correlated with these metabolites. *Anaerostipes* and *Lachnospiraceae-UCG-006* were negatively correlated with indole and indole-3-acetic acid, and positively associated with SCFAs including butyric acid and propionic acid. For metabolites-skin-proteins associations (**Figure 7B**), butyric acid correlated positively with AMPK pathway proteins (Ulk1, Pfkfb4, Scd3), and negatively with p38 MAPK proteins (Map3k6, Map3k7) and PI3K-Akt proteins (Efna1, Zfp36). Indole and indole-3-acetic acid correlated positively with AMPK proteins (Ulk1, Pfkfb4), and negatively with p38 MAPK proteins (Kcnj8, Map3k6, Map3k5, Mapk14) and PI3K-Akt proteins (Efna1, Col6a1, Col6a2, Col6a3). Conversely, arachidonic acid, leukotriene B4, 3-oxohexadecanoic acid, 5-oxooctadecanoic acid, and deoxycholic acid correlated positively with the p38 MAPK and PI3K-Akt proteins, and negatively with the AMPK proteins (*P<0.05*). Overall, these correlations suggest potential associations among gut microbiota, metabolites, and skin proteins in response to JF treatment.

### 3.8 JF upregulates p-AMPK and downregulates p-p38 MAPK, p-PI3K, and p-Akt in the skin of urticaria model rats

To biochemically validate above-mentioned proteomic findings, IHC and WB assays were performed to quantify phosphorylated and total protein levels of AMPK, p38 MAPK, PI3K, and Akt in rat skin samples (**Figures 8A, B**).

**Figure 8:**
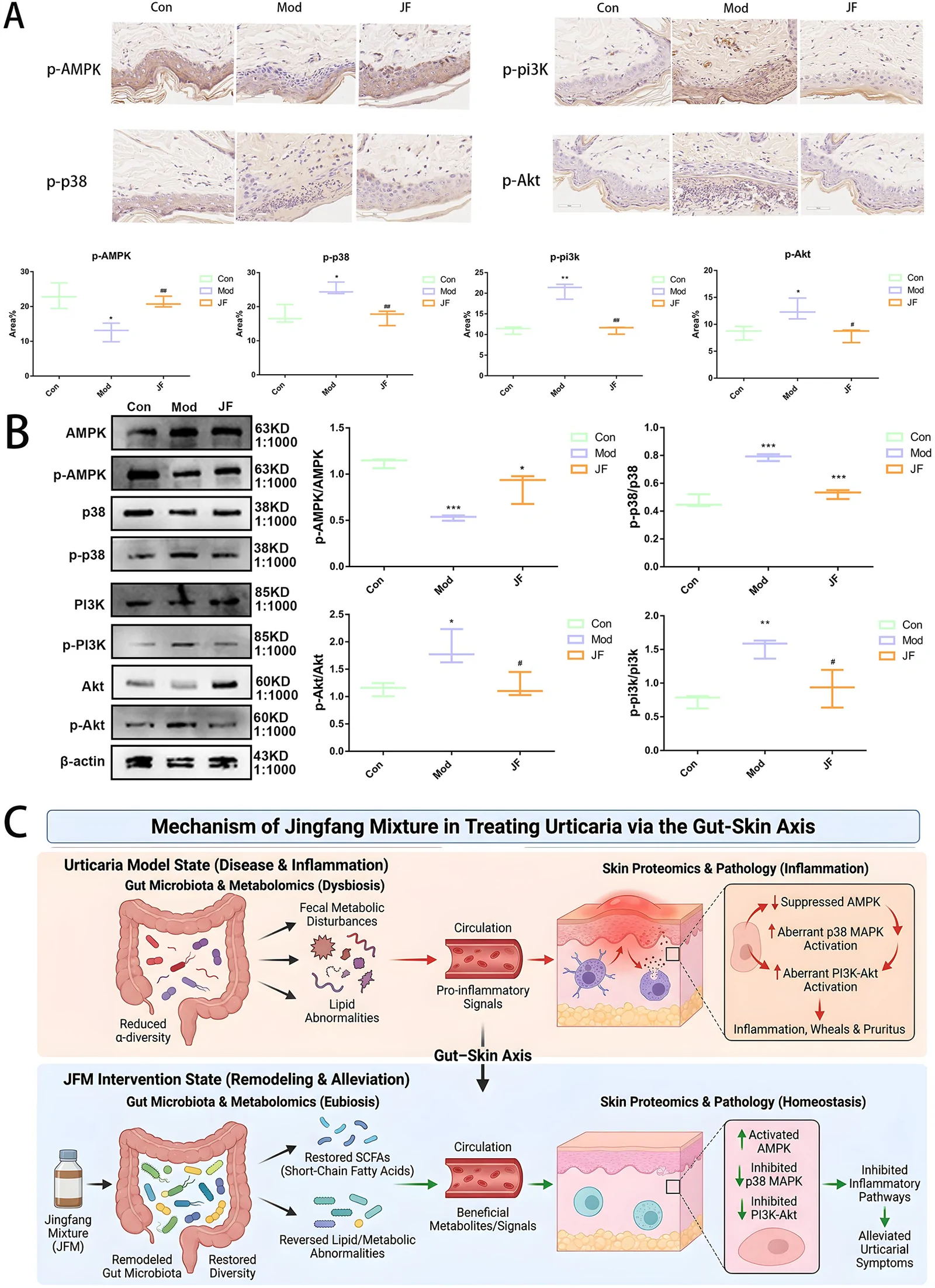
JF regulates the phosphorylation levels of AMPK/p38 MAPK/PI3K-Akt pathway-related proteins in the skin tissue of urticaria model rats and a schematic diagram of the mechanism. **(A)** Representative IHC images of p-AMPK, p-p38 MAPK, p-PI3K, and p-Akt in the skin tissue of rats in each group (scale bar=50 μm); and quantitative analysis of IHC integrated optical density (IOD) (n=3). **(B)** Western blot bands for phosphorylated and total proteins; and quantitative analysis of p-AMPK/AMPK, p-p38/p38, p-PI3K/PI3K, and p-Akt/Akt ratios (n=3). **(C)** Schematic diagram illustrating the mechanism by which JF alleviates urticaria-like inflammation by regulating the AMPK/p38 MAPK/PI3K-Akt pathway associated with the gut-skin axis. One-way ANOVA with Tukey’s/Dunnett’s *post hoc* test after normality/variance tests (Kruskal-Wallis with Dunn’s test for non-normal data). Exact P values in **Supplementary Table 5**. *P<0.05, **P<0.01, ***P<0.001 vs. Con; ##*P<0.01*, ###*P<0.001* vs. Model.

IHC results demonstrated moderate positive staining for p-AMPK in the Con group, whereas p-p38 MAPK, p-PI3K, and p-Akt exhibited only faint immunoreactivity. In the model group, the staining area and signal intensity of p-AMPK were significantly reduced (*P<0.05*). By contrast, immunoreactivity of p-p38 MAPK, p-PI3K, and p-Akt were substantially augmented (*P<0.05*), predominantly localized within the cytoplasm of epidermal keratinocytes and dermal inflammatory-cell-infiltration zones. JF intervention reversed these changes: p-AMPK increased (*P<0.01*), and p-p38 MAPK, p-PI3K, and p-Akt decreased (*P<0.05*) (**Figure 8A**). Western-blot quantification corroborated IHC observations (**Figure 8B**). The p-AMPK/AMPK ratio was significantly reduced in the model group compared with the Con group (*P<0.01*) and was restored by JF treatment (*P<0.05*). Conversely, p-p38/p38, p-PI3K/PI3K, and p-Akt/Akt ratios were drastically increased in the model group (*P<0.01*), which were significantly reduced following JF administration (*P<0.01, P<0.05*, and *P<0.05*, respectively). Together, IHC and WB results confirm that JF upregulates p-AMPK and downregulates p-p38 MAPK, p-PI3K, and p-Akt in the skin of urticaria-like model rats. This reciprocal regulatory pattern targeting the AMPK/p38 MAPK/PI3K-Akt signaling axis likely contribute to JF-conferred protective effects against urticaria-like allergic inflammation in this rat model.

## 4 Discussion

Second-generation H1 antihistamines cannot achieve sufficient symptom remission for all urticaria patients, highlighting the necessity to dissect systemic pathological mechanisms beyond cutaneous mast-cell activation.

Cumulative evidence supports gut-skin crosstalk as an important contributor to urticaria pathogenesis; nevertheless, whether JF-driven alterations in gut microbial composition and fecal metabolic profiles are functionally linked to cutaneous inflammatory and immunometabolic signaling remains incompletely elucidated. In the present study, we integrated gut-microbiome, fecal-metabolome, and skin-proteome datasets in an OVA/Al(OH)_3_-induced rat model of urticaria-like allergic inflammation to examine this potential cross-compartment relationship.

Pathogenically, urticaria is characterized by mast-cell activation and type-2 immune responses, which promote histamine release and downstream inflammatory cascades (Church et al., 2018; Pala et al., 2025). Furthermore, accumulating evidence implicates Th17-associated inflammation in chronic urticaria, consistent with the elevated IL-17 observed in our animal model (Chen et al., 2018). The therapeutic activities of JF manifested across three interconnected biological layers. First, JF reduced IgE and several inflammatory cytokines, including IL-4, IL-6, TNF-α, and IL-17, suggesting effects on both allergic and inflammatory responses. Second, intact intestinal-barrier function is indispensable for sustaining host immune homeostasis. Once intestinal barrier integrity is compromised, endotoxins and microbial products can translocate into systemic circulation and further amplify inflammatory responses (Neurath et al., 2025; Xu et al., 2025; Zang et al., 2026). JF improved intestinal barrier-associated pathological abnormalities and corrected gut dysbiosis. Reduced serum DAO and D-LAC, together with improved colonic histopathology indicate ameliorated intestinal-barrier dysfunction, consistent with prior reports linking intestinal hyper-permeability and urticaria disease severity (Zhu et al., 2024; Ko et al., 2025). Third, JF restored fecal metabolome, raising SCFA and indole metabolites while diminishing pro-inflammatory lipid mediators and bile-acids species. Mechanistically, butyrate modulates T-cell differentiation and represses IL-4 and IL-17 production via G protein-coupled receptors or histone deacetylase inhibition (Xiao et al., 2026; Yang et al., 2026); indole-derivatives metabolites reinforce intestinal-barrier and foster host immune tolerance (Zhang et al., 2026a, Zhang et al., 2026b). Arachidonic acid is a precursor of inflammatory lipid mediators, including LTB4, which can promote inflammation through leukotriene-receptor signaling (Izaki et al., 1996; Giraud, 2026).

Chemical profiling of JF identified multiple major bioactive constituents including terpenoids, flavonoids, and alkaloids. Previous pharmacological investigations have verified that these compounds possess anti-inflammatory and immunomodulatory activities, and several of them are capable of regulating AMPK signaling (Hao et al., 2024). These findings support the hypothesis that the multi-target effects of JF on the gut-skin axis may be attributed, at least in part, to these bioactive constituents. In addition, proteins associated with mitochondrial respiratory-chain complex I were enriched among the DEPs following JF treatment, suggesting alterations in mitochondrial energy metabolism-related processes. However, mitochondrial alterations directly contribute to enhanced AMPK phosphorylation remains to be clarified. This suggests that JF may improve skin mitochondrial energy metabolism upstream of AMPK activation (Zhang and Zhang et al., 2015; Hozumi et al., 2023). As a central hub coordinating cellular energy metabolism and inflammatory control, activated AMPK suppresses PI3K-Akt cascades, curtails pro-inflammatory cytokine secretion, and facilitates fatty-acid oxidation (Chen et al., 2025, Chen et al., 2026). In this study, JF treatment increased AMPK phosphorylation while decreasing phosphorylation levels of p38 MAPK, PI3K, and Akt. These findings were concordantly supported by proteomics, GSEA enrichment, and immunohistochemical results. Multi-omics correlation analyses revealed coordinated changes across gut microbiota-metabolite-skin signaling association. While our multi-omics data suggest an association among gut microbiota, metabolites, and skin signaling, causal relationships remain to be established. Future mechanistic studies could adopt experimental frameworks analogous to polysaccharide research: integrating individual gut-microbial composition, metabolomic profiles, and host genetic backgrounds to construct personalized computational gut-ecosystem models, so as to validate the causal “microbiota-metabolite-host” regulatory chain (Chen et al., 2026). Such validation experiments were not implemented in the current work due to constraints of time and research resources.

Beyond individual molecular targets, these findings reinforce that urticaria is associated with systemic gut-skin inter-organ communication and uncover a potential functional link between SCFA bioavailability and cutaneous AMPK activity. This interconnection bridges gut microbial metabolism and skin immunometabolic signaling and provides quantifiable experimental evidence for this previously conceptually described gut-skin-axis phenomenon. For TCM research, our work offers a biological interpretative framework for understanding multi-target therapeutic mechanisms of classical wind-dispersing herbal formulas that act simultaneously on intestinal and cutaneous compartments. The multi-omics approach adopted here, linking 16S rRNA data, metabolomic profiles, and proteomic changes, enabled us to identify coordinated associations among gut ecological shifts, metabolic outputs, and skin signaling events. This integrative strategy is particularly suited for studying complex systems like the gut-skin axis, where single-layer analyses would fail to capture the inter-compartment crosstalk. It should be noted that proteomics and Spearman correlation analyses were performed with n = 3 biological replicates for each group. This relatively small sample size compromises statistical power and may generate unstable correlation estimates. Hence, our omics-derived and pathway-level findings should be regarded as exploratory and hypothesis-generating; follow-up studies with enlarged sample sizes are required for independent validation.

Collectively, our results suggest JF merits further pre-clinical and translational evaluation as a multi-target therapeutic candidate for urticaria, although rigorous clinical validation is still indispensable. Future investigations could test the therapeutic potential of SCFA supplementation, AMPK activation, and microbiota-directed adjuncts interventions. Fecal SCFA concentrations, circulating intestinal-barrier markers, and cutaneous p-AMPK abundance could serve as candidate biomarkers for future mechanistic and translational studies. Several limitations should be acknowledged. First, this animal model recapitulates urticaria-like acute allergic inflammation but cannot the chronic, relapsing course of human CSU. Whether identical molecular mechanisms operate in chronic urticaria or human patients awaits further verification. Only male rats were enrolled in this experiment, which restricts the generalizability of our conclusions to female animals and human subjects of both genders. Follow-up animal studies incorporating both male and female rats are warranted to evaluate whether the gut-skin-axis-related mechanisms uncovered herein exhibit sex-dependent characteristics. Second, 16S rRNA sequencing outputs reflect bacterial relative abundance, which can be confounded by global shifts in gut-microbial community structure. Negative-control samples were not included during the original 16S rRNA sequencing workflow, which may compromise detection accuracy for low-biomass contaminating sequences; future sequencing projects should incorporate negative controls to monitor contamination. Absolute microbial quantification via qPCR was not performed in this study. In addition, correlation analyses only support associative relationships rather than causal inference. Causal-validation experiments, such as fecal-microbiota transplantation and identification of cell-surface receptors mediating SCFA-triggered skin AMPK activation, are urgently required. Third, intestinal-barrier integrity was assessed via circulating DAO/D-LAC biomarkers and colonic histology. Direct detection of tight-junction protein abundance and functional intestinal-permeability assays were not conducted. Follow-up investigations incorporating these readouts are needed to verify whether the observed gut-skin-axis alterations constitute a true causal pathogenic cascade. Fourth, JF is a composite multi-herbal formula, and the specific bioactive compound(s) responsible for its *in-vivo* protective effects remain unidentified. Further experiments testing individual herbal components and compound combinations are required to pinpoint the key pharmacologically active constituents. Moreover, although ELISA kits were manufacturer-validated for rat serum specimens, detailed cross-reactivity data were not provided by vendors, and independent cross-reactivity validation was not performed in our laboratory. This caveat should be considered when interpreting absolute quantitative values for measured analytes. As mentioned above, proteomics and Spearman correlation analyses were conducted with merely n = 3 biological replicates for each experimental group. Limited sample size reduces statistical power and may produce unstable estimates. Therefore, multi-omics-derived findings are exploratory and hypothesis-generating in nature; independent validation with larger sample cohorts is required in future work.

## 5 Conclusion

This pre-clinical study provides evidence that JF alleviates OVA/Al(OH)_3_-induced urticaria-like allergic inflammation in rats by reducing skin lesions and systemic inflammatory responses and ameliorating intestinal barrier-associated abnormalities. JF treatment was accompanied by coordinated changes in gut microbial composition, fecal metabolism, and cutaneous AMPK/p38 MAPK/PI3K-Akt signaling. The integrated multi-omics data support a potential gut microbiota-metabolite-skin signaling network associated with the anti-inflammatory effects of JF and provide a basis for further mechanistic and translational investigation.

## Data Availability

The data analyzed in this study is subject to the following licenses/restrictions: The datasets used and/or analysed during the current study are available from the corresponding author on reasonable request. Requests to access these datasets should be directed to Xijun Wang,xijunw@sina.com.
