# Supplementary material for: Multi-omics analysis revealed that Jingfang Mixture alleviates inflammation in an OVA/Al(OH)3-induced urticaria-like rat model by regulating the AMPK/p38 MAPK/PI3K-Akt pathway associated with the gut-skin axis
Source: Front Cell Infect Microbiol. 2026 Oct 29;16:1973292. doi: 10.3389/fcimb.2026.1973292 (PMC13754694; doi:10.3389/fcimb.2026.1973292)
Supplement: Supplementary file 1 [file Table1.docx]

Supplementary Table 1	Kit lot number and other parameters

| Kit | Catalog No. | Lot No. | Species Reactivity | Detection Range |
| --- | --- | --- | --- | --- |
| HIS | JM-01941R1 | 20250523 | Rat | 1.5-60μg/L |
| IL-4 | JM-01598R1 | 20250523 | Rat | 3-150pg/L |
| IL-6 | JM-01597R1 | 20250523 | Rat | 3-150pg/mL |
| IL-17 | JM-01495R1 | 20250523 | Rat | 0.2-20ng/L |
| Ig-E | JM-01470R1 | 20250523 | Rat | 0.1-4μg/L |
| TNF-α | JM-01587R1 | 20250523 | Rat | 10-500ng/L |
| DAO | JM-02209R1 | 20250523 | Rat | 5-180pg/L |
| D-LAC | JM-105017R1 | 20250523 | Rat | 30-1500μg/L |
