# Supplementary material for: Multi-omics analysis revealed that Jingfang Mixture alleviates inflammation in an OVA/Al(OH)3-induced urticaria-like rat model by regulating the AMPK/p38 MAPK/PI3K-Akt pathway associated with the gut-skin axis
Source: Front Cell Infect Microbiol. 2026 Oct 29;16:1973292. doi: 10.3389/fcimb.2026.1973292 (PMC13754694; doi:10.3389/fcimb.2026.1973292)
Supplement: Supplementary file 2 [file Table3.docx]

Supplementary Table 3 List of abbreviations

| **abbreviations** | **Full name** |
| --- | --- |
| AMPK | AMP-activated protein kinase |
| Akt | protein kinase B |
| ASV | amplicon sequence variant |
| BSA | bovine serum albumin |
| CST | Cell Signaling Technology |
| DAB | 3,3′-diaminobenzidine |
| DAO | diamine oxidase |
| D-LAC | D-lactic acid |
| DIA | data-independent acquisition |
| ECL | enhanced chemiluminescence |
| ELISA | enzyme-linked immunosorbent assay |
| GO | Gene Ontology |
| GSEA | gene set enrichment analysis |
| H&E | hematoxylin and eosin |
| HRP | horseradish peroxidase |
| IHC | immunohistochemistry |
| IOD | ntegrated optical density |
| JF | Jingfang Mixture |
| KEGG | Kyoto Encyclopedia of Genes and Genomes |
| LEfSe | linear discriminant analysis effect size |
| OVA | ovalbumin |
| PI3K | phosphoinositide 3-kinase |
| PVDF | polyvinylidene fluoride |
| RIPA | radioimmunoprecipitation assay |
| SCFA | short-chain fatty acid |
| SDS-PAGE | sodium dodecyl sulfate-polyacrylamide gel electrophoresis |
| TCM | traditional Chinese medicine |
| TNF-α | tumor necrosis factor-α |
| WB | Western blot |
