# Supplementary material for: Multi-omics analysis revealed that Jingfang Mixture alleviates inflammation in an OVA/Al(OH)3-induced urticaria-like rat model by regulating the AMPK/p38 MAPK/PI3K-Akt pathway associated with the gut-skin axis
Source: Front Cell Infect Microbiol. 2026 Oct 29;16:1973292. doi: 10.3389/fcimb.2026.1973292 (PMC13754694; doi:10.3389/fcimb.2026.1973292)
Supplement: Supplementary file 3 [file Table4.docx]

Supplementary Table 4 JF2 vs JF4(JF6) effect sizes and 95% confidence intervals

| 21d | Compare | Mean difference (JF2 − JF4/ JF6) | Combined standard deviation SP | Standard error SE*SE* | Mean difference 95% CI | Cohen’s d | Hedges’ g | Hedges’ g 95% CI |
| --- | --- | --- | --- | --- | --- | --- | --- | --- |
| Scratching number | JF2 vs JF4 | 6 | 5.43 | 4.43 | (-6.31, 18.31) | 1.105 | 0.884 | (-0.834, 2.602) |
| Scratching number | JF2 vs JF6 | -2.33 | 6.65 | 5.43 | (-17.39, 12.72) | -0.351 | -0.281 | (-1.894, 1.332) |
| Erythema area of skin | JF2 vs JF4 | -1.111 | 3.32 | 1.56 | (-4.427, 2.205) | -0.335 | -0.319 | (-1.249, 0.611) |
| Erythema area of skin | JF2 vs JF6 | -3.889 | 3.16 | 1.49 | (-7.046, -0.732) | -1.231 | -1.172 | (-2.179, -0.165) |
| Mast cell number | JF2 vs JF4 | -5.334 | 3.16 | 1.49 | (-8.495, -2.173) | -1.687 | -1.607 | (-2.683, -0.531) |
| Mast cell number | JF2 vs JF6 | -4.667 | 2.96 | 1.39 | (-7.622, -1.712) | -1.578 | -1.503 | (-2.561, -0.445) |
| Histologic scores | JF2 vs JF4 | -0.211 | 0.279 | 0.131 | (-0.490, 0.067) | -0.758 | -0.722 | (-1.678, 0.234) |
| Histologic scores | JF2 vs JF6 | -0.379 | 0.205 | 0.1 | (-0.591, -0.167) | -1.852 | -1.758 | (-2.896, -0.620) |
